# A new comprehensive model for Continuous Professional Development

**DOI:** 10.1080/13814788.2016.1256998

**Published:** 2016-12-20

**Authors:** Niels Kristian Kjaer, Marianne Vedsted, James Høpner

**Affiliations:** ^a^ Research Unit of General Practice, Institute of Public Health, University of Southern DenmarkOdenseDenmark; ^b^ The Specialty Training in Family Medicine, Institute of Public Health, Aarhus UniversityAarhusDenmark; ^c^ Association of General Practitioners, Department of Continuing Medical EducationCopenhagenDenmark

**Keywords:** General practice, educational needs assessment, curriculum, continuing medical education

## Abstract

**Background:** It is generally agreed that continuing professional development (CPD) for GPs is important for quality of care. Internationally, however, different approaches to identify the learning objectives and the CPD content have been chosen.

**Objectives:** To improve GPs’ CPD in Denmark we explore how general practitioners’ (GPs) self-experienced learning needs can be combined with learning needs experienced from a societal perspective and still make sense for GPs.

**Methods:** We performed a multi-dimensional learning needs analysis with a modified Delphi method in a participatory action research set-up. Twenty practice-based small learning groups and a group appointed by the Danish public health service were asked to identify learning needs with the Danish family medicine curriculum as reference. Then we asked a group of GP researchers and hospital consultants, a group of GPs with interests in narrative, person-centred medicine and a group of GP educators, and administrative staff, to triangulate the initial findings.

**Results:** We identified educational themes through a defined collaborative consensus oriented process. Examples of themes are the diagnostic challenge, care for patients with multi-morbidity, elderly patients and children. Due to variation in requested learning objectives, the identified themes do not cover all relevant areas for CPD training. The identified themes will only make sense if seen as supplementary to other CPD activities based on GPs individual needs analyses.

**Conclusion:** It is possible to identify prioritized educational themes for GPs through a process involving the majority of stakeholders. Nevertheless, CPD should also include activities based on individual needs analysis.

KEY MESSAGES

- When developing a continuing professional development programme for GPs, educational themes can be identified by a multi-dimensional needs analysis involving different stakeholders.
- GPs might benefit from activities based on a consensus-based curriculum combined with an individual needs analysis, thus exposing individual GPs to both recognized and non-recognized relevant learning needs.

## Introduction

A comprehensive continuing professional development (CPD) programme for general practitioners (GPs) is essential for developing and maintaining high professional standards in general practice.[1] The way such a CPD programme is organized seems to make a difference in relation to the expected outcome.[2,3] We know the highest impact on clinical performance is achieved if the CPD activities contain a combination of an update of knowledge and interactive learning.[2] Educational research also emphasizes the importance of individual needs analysis and self-assessment,[4,5] the importance of context in learning and the importance of providing strategies on how to implement new knowledge into daily clinical practice.[5–8]

In Denmark, as in many other countries, there is in an interest in how to create a more effective and efficient CPD programme for GPs. Danish family medicine was recognised as medical specialty in 1993 and the Danish specialist-training programme lasts for five years.[9]

Only specialists in family medicine can work as GPs. Danish GPs are frequent users of voluntary, accredited CPD activities. Their CPD choices seem to be motivated by topics, which the GPs themselves believe will strengthen their professional capacity and prevent burnout.[3] There is no formal GP revalidation system. The Danish GP CPD programme is shown in Box 1.

Box 1. The Danish Continuing Professional Development (CPD) programme for General Practitioners (GPs).

The Danish CPD programme for GPs is voluntary, and has no formal requirements regarding attendance and content.

- It is based on accredited activities remunerated up to approximately EUR 1800 per year.
- There is no funding for non-accredited CPD activities.
- Courses arranged by the pharmacological industry are not accredited.
- Individual GPs decide which of the accredited CPD activities they wish to attend.
- CPD activities are often a combination of practice-based small group learning (PBSGL) and traditional CPD courses.

The appropriateness and efficiency of the Danish voluntary programme is debated and the need for mandatory elements proposed.[10] It has been suggested that all GPs should participate in a collective learning programme to ensure a minimum level of competences. Such an initiative has to make sense for GPs in order to be embraced and in order to have impact in practice.[3,11] GPs’ selection of courses and training programmes are by no means random.[3] It seems appropriate to introduce mandatory elements in CPD training only with prior careful and deliberate considerations.

Internationally, a variety of approaches have been tried to detect GPs learning needs.[12,13]

An appraisal process can, if properly used, induce reflection and assist in the choice of CPD activities.[13] Experiences from UK, however, indicate that appraisal is not experienced as beneficial by all GPs.[14] A study has shown that Danish GPs are reluctant towards an appraisal process but are instead open to a system in which their voluntary CPD programmes were supplemented with CPD activities based on a consensus-based curriculum.[15]

The Danish Regions (responsible for the national health services including the provision of general practice) and the Association of General Practitioners in 2014 entered into an agreement that aligned the incentives to participate in CPD.[16] The existing voluntary, individually based CPD programme in Denmark was extended with recommended and prioritised structured and systematic CPD activities.

In this article, we explore whether it is possible to identify and prioritise relevant objectives for these new systematic CPD activities, which would make sense both to GPs’ self-experienced learning needs and to the learning needs seen from a societal perspective.

## Methods

### Study design

**Figure 1.**
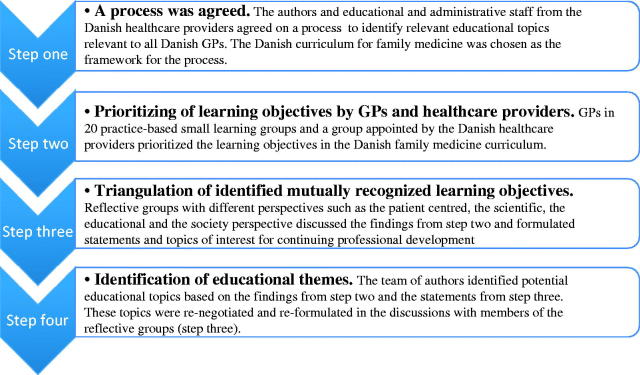
Modified Delphi method in a participatory action research set-up to identify mutual learning needs and to identify educational objectives for Danish GPs.

We used a modified Delphi method in a participatory action research set-up.[17] The process, consisting of four consecutive steps of data collection and analysis, is illustrated in Figure 1.

### Step 1: Consensus meeting on study design

The first step was a meeting in November 2014 between GP educators from the Association of General Practitioners and GP educators and administrative staff employed by the Danish Regions in order to agree on how to define a feasible design for the need analysis. The national curriculum of Danish family medicine specialist training was used as a framework.

### Step 2: Generating learning objectives

#### Practice-based small learning groups

In the second step, five regional GP-educators from different parts of Denmark were asked to nominate 20 practice-based small learning groups (PBSLG) to take part in a needs analysis. These PBSLG consisted of 4–16 GPs. A priori, the curriculum of Danish family medicine was divided into five domains to make it operational for CPD-planning.[10] Each PBSLG was asked to analyse one of these five domains, equivalent to one fifth of the entire curriculum.

At the individual PBSLG meetings, in February 2015 members discussed the learning objectives from the curriculum, first in pairs and then in plenum, thereafter rating the learning objectives in relation to perceived relevance and priority for their future CPD.

Two of the authors coded the ratings from the groups on a 0–20 scale. The average score for the rating of the objectives and 95% confidence intervals was calculated in STATA 13. The objectives with a score on the 75th centile or above were defined as ‘highly prioritized’ by the GPs.

After the data collection from the PBSLGs, we realized that the output from some of the groups did not strictly follow the framework applicable in setting ratings. These groups instead reported a combination of quantitative ratings of the learning objects and qualitative statements. It was therefore necessary to recode the outcome from these groups. Two of the authors recoded the ratings and statements from these groups independently. Statements such as ‘This learning objective is the most important of all’ were rated with the score 20. ‘This learning objective is pretty important and should be prioritized’ was rated with the score 10. ‘This objective is important, but we don’t give it top priority’ was rated with the score 5. ‘This objective is not relevant’ was rated with the score 0. In case of disagreement among raters, the lowest score was used.

### Regional healthcare administrations

Eleven representatives from different regional healthcare administrations appointed by the Danish Regions discussed February 2015 the same curriculum and identified objectives they believed should be prioritized for structured CPD training from a societal perspective.

The Danish Regions group rated the learning objectives into two categories: ‘high’ versus ‘low’ priority in relevance for CPD for GPs.

### Matching learning objectives

The authors matched the objectives that the PBSLGs rated highest with the prioritized list of objectives from the Danish Regions. Mutually agreed learning objectives were thus identified. The remaining prioritized but not agreed objectives were also recorded, for later analysis.

### Step 3: Triangulation of learning objectives

The third step was a triangulation of the agreed learning objectives. The findings from step two were discussed in three different reflective groups (RG).

- Group A was a group researchers appointed by the four Danish university departments of family medicine and hospital consultants appointed by the Danish Regions.
- Group B was a group of GPs who had a special interest in a patient-centred approach and in the humanistic dimension of the GP role. The members of this group were nominated by the Danish College of General Practitioners.
- Group C was a group of GP educators and administrative staff employed and appointed by the Danish Regions.

The qualitative statements and conclusion from groups A, B and C were transcribed and analysed by the three authors and agreement negotiated.

### Step 4: Formulation of educational themes

In step four, the authors condensed the data from the previous steps and presented it to groups B and C. In follow-up discussions with the groups, the educational themes were negotiated and formulated. There was focus on obtaining consensus among the participating collaborators.

It was not possible to gather group A in the follow-up process. The participants in group B were paid a fee to participate, the rest were paid only transportation expenses.

## Results

### Mutually agreed learning objectives (step 2)

All the 20 nominated PBSLGs with approximate 150 GPs, and the 11 persons from the healthcare providers participated in the needs analysis and delivered data. The GPs and the administrators agreed on objectives from 25 out of 84 learning goals listed the Danish family medicine curriculum. A list of the mutually agreed learning objectives (in English) and the data set including the Danish family medicine’s objectives with the ratings from GPs and administrators (in Danish) are attached as a supplement. The GP rating of the learning objectives showed scores from 0 to 20 with a mean score of 5.3 (95% CI: 4.8–5.8). The 75th centile score was 7.5. The 90th centile score was 11.25.

### Statement from the triangulation process (step 3)

Results and statements from the discussions in groups A, B and C were condensed into the following statement:

- The diagnostic process is a major challenge. GPs have to detect the few rare and serious diseases among a majority of less ill patients with weak and non-specific symptoms. GPs work in the pre-diagnostic part of the healthcare system and need multiple paradigms of understanding to meet patients’ needs. Feasible and relevant use of scans, X-ray and laboratory tests are important. Over-diagnosis is an important issue.
- There seemed to be issues around both over-diagnosis and under-diagnosis of mental diseases as well as inaccurate use of medication.
- In caring for patients with chronic diseases, the ability to organize and lead a GP surgery was ranked as an important element that could not be separated from medical competence.
- The treatment of elderly patients was regarded as complex and could be optimized.
- It was also important to be able to diagnose and treat patients with multi-morbidity and patients with medically unexplained symptoms
- In general, the GPs found that education should be prioritized in topics where GPs played an important role in patient care, and where GPs played a pivotal role in solving issues particularly when more specialized treatment was not available.
- A person-centred approach and an ability to understand the patient’s world and beliefs was considered a prerequisite for proper care in general practice.
- The ability to use information technology in patient care was mentioned as being of growing importance.

### Educational themes (step 4)

Eleven educational themes were identified and were prioritized (Box 2). The themes should combine specific medical textbook knowledge with the experienced-based knowledge among participating GPs. They should also embed other central skills, such as communicative skills, the ability to collaborate with peers, staff, hospital consultants and community workers, and the ability to lead and organize a GP practice.

## Discussion

### Main findings

We have described a model for need assessment and identified educational themes suitable for a CPD-programme for Danish GPs, such as the diagnostic challenge, care for patients with multi-morbidity, elderly patients, and children, mental health and palliative care. The themes seem to make sense both for GPs and society. Due to variation in the reported learning needs, we also found that the identified themes did not cover all relevant learning needs. The suggested themes should therefore be seen as a consensus based supplement to CPD-programmes, which also include activities based on individual need analysis.

Box 2. The educational topics.

**The diagnostic challenge**

- Early detection of serious diseases such as cancer in relation to unspecific symptoms commonly presented to the GP such as tiredness or dizziness, without introducing unnecessary anxiety or over-diagnosing and without excessive use of referrals.
- Proper and rational use of X-rays, scans, lab tests and information technology.
- Problems related to over-diagnosing and under-diagnosing of common diseases.
- Care for patients with multiple symptoms but without available diagnosis (medically unexplained symptoms).
- How to use communication and the ability to provide active listening as a tool to qualify the diagnostic process.

**Problems related to children and teenagers**

- The sick child.
- The child with pain complaints.
- Problems related to behaviour disorders, teenage depressions and self-inflicted harm.
- The child at risk, including the child in the sick family.

**Problems related to the elderly patient**

- Somatic problems and symptoms, problems as they are presented to the GP including problems related to multi-morbidity, polypharmacy and side effects.
- Psychological and cognitive problems, as they are presented to the GP, such as sorrow, confusion, depression, dementia and medical abuse.

**Patients with several chronic diseases**

- Care for patients with multi-morbidity including how to maintain a person-centred perspective and how to handle multiple guidelines with contradictory recommendations.

**Psychological and psychiatry problems as they are presented to the GP**

- How to handle and differentiate between, stress, grief, existential problems, abuse and depression.
- **How to handle psychiatric disorders as they are presented to the GP.**

**Care for dying patients**

**Neurological symptoms as they are presented to the GP**

**Skin symptoms as they are presented to the GP**

**Proper and rational use of antibiotics**

**Identification of severe eye diseases**

**Symptoms from joints, muscles and tendons**

### Strengths and limitations

The 20 participating learning groups represented only approximately 10% of all PBSLGs in Denmark. Furthermore, approximately 5% of GPs are not members of any group. We had to include a strategy of recoding qualitative statements into a score, which includes a risk of researcher bias influencing the data interpretation. However, we attempted to minimize bias by enrolling authors with different educational backgrounds and by deliberately engaging peers and stakeholders with very different perspectives. We negotiated a mutual understanding in a participatory action research setup in which the participants were not only informants but also collaborators.[17] By this triangulation of views between GPs and Danish Regions with discussions among researchers, hospital consultants, respected GPs and employees from the administrative staff we assume the degree of representativeness of the findings is acceptable.

In step 2, we chose the 75th percentile as cut-off for the GPs ratings based on an estimate. It resulted in an equal amount of learning objectives prioritized by the GPs and the contractors. The majority of objectives included in the consensus curriculum scored above the 90th centile.

We identified learning needs by allowing the participants to evaluate GPs’ learning needs in relation to the national curriculum of Danish family medicine specialist training. The Danish curriculum has been reviewed based on years of experience; it has been aligned with relevant international curricula, included patients involvement, and it focuses on general competencies not easily out-dated.[18] We assumed it was a reliable framework for GP competencies.

### Comparison with existing literature

European CPD programmes are organized differently, covering the scoop from voluntary CPD to partly mandatory programmes with or without revalidation.[10] We also see different approaches to learning need identification.[3] Britain has implemented an appraisal system, where each GP has to define own learning needs in collaboration with an appraiser. The acceptance of this system varies and it is considered labour intensive by some GPs.[14] Danish GPs do not request external assistance in CPD.[15] The Danish reluctance toward appraisal can be challenged by findings in the literature.[13] But our study offers an alternative consensus-based mixed top-down, bottom-up approach to learning needs analysis in line with Danish GPs’ preferences.[15] This approach may be of interest in European countries discussing how to address GPs’ learnings needs.[5,12] Several of our identified themes have also been identified by others as important for CPD e.g. the diagnostic challenge and multi-morbidity.[19,20]

In our set up, we have focused on how to detect learning objectives relevant for the majority of well-qualified Danish GPs. In any population of professionals there can be assumed to be a minority of persons who are neither fit nor qualified to practise.[21] These GPs cannot be reached by general CPD programmes. We need specific and special programmes to identify and assist this small group of GPs in professional crises.[21] We therefore think general CPD programmes should aim after the learning needs of majority of well-qualified GPs.

### Implications for research and/or practice

We identified objectives, where there were blatant disagreements in prioritization. In a future study, these different prioritizations should be explored. Patients have not been directly involved in our needs analysis, further studies could analyse how meaningful patient involvement could be obtained. A comprehensive evaluation of the suggested educational themes is necessary to adjust and redefine the objectives in future CPD programmes.

## Conclusion

It is possible to identify a group of highly prioritized educational themes for Danish GPs through a process involving the majority of stakeholders. The themes seem to make sense both to GPs’ self-experienced learning needs and to the learning needs seen from a societal perspective. However, a CPD programme would probably benefit from a combination of activities based on a consensus-based curriculum and activities based on an individual needs analysis.

## Supplementary Material

Supplemental_material
