# Supplementary material for: A new comprehensive model for Continuous Professional Development
Source: Eur J Gen Pract. 2016 Dec 20;23(1):20–6. doi: 10.1080/13814788.2016.1256998 (PMC5774289; doi:10.1080/13814788.2016.1256998)
Supplement: Supplemental_material [file igen_a_1256998_sm5031.pdf]

Appendix

The learning objectives from the Danish Curriculum in family medicine training were prioritised by 20 practice based small learning groups and by staff from the healthcare services. The prioritised learning objectives listed below were obtained by combining objectives with a GP score above 7.5 point with the high prioritised objective of the health care service.

Domain one

Patients with symptoms from heart circuit, bowel and musculoskeletal system, and serious acute conditions.

Diagnose, treatment and control of dyspnoea, allergy, asthma, respiratory tract infections.

Diagnose, treatment and make relevant visitation to specialists in patients with fatigue.

Diagnose, treatment, and visitation for patients with blood in stools or stool changes and serious gastrointestinal disorders.

Rational choices of antibiotic treatment concerning resistance pattern, resistance development and hypersensitivity

Be able to initiate acute treatment (oxygen, intravenous hydration/fluids, Trendelenburg, resuscitation)

Perform the social and occupational medicine aspects of chronic pain from musculoskeletal system.

Perform primary diagnosis, treatment and visitation of patients with acute or chronic joint pain.

Domain two

Patients with symptoms from CNS, sense bodies, skin, and patients with mental diseases.

Handle patients with neurological symptoms.

Examine, visitation and handle patients with paresis, staples, paraesthesia, sensory loss, neurogenic pain and cognitive disorder.

Know the rules regarding driving license and neurological diseases.

Diagnose, treatment and visitation of patients with headache, for example: tension headache, medication induced headache, migraine, Intracranial processes.

Diagnose, treat and visitation of patients with dizziness of both somatic and psychological causes.

Handle patients with skin symptoms. Could distinguish the harmless and transient skin manifestations from those requiring treatment and further investigation/visitation in both children and adults, including the skin manifestations as part of an underlying systemic disease.

Diagnose, visitation and treatment of patients with acute psychosis. Could initiate and make the necessary

attestation regarding involuntary visitation to hospital in patients with psychosis.

Diagnose, and visitation of patients with eating disorders and possible personality disorders.

Diagnose, handle, treatment and visitation of patients with depression, anxiety disorders and stress related disorders.

Domain three.

Patients with symptoms from urinary disorders and reproduction system and health problems in children.

Deal with children with fever in: Upper airways/tract infections, otitis, tonsillitis, pneumonia, gastro-enteritis, pseudo croup, recurrent infections and sustained fever.

Identify symptoms of serious infectious diseases requiring hospitalisation.

Perform primary elucidation and visitation of chronic diarrhoea, urinary tract infections, involuntary nightly urination, retentio and torso testis, abdominal pain, kidney diseases and orthopaedic diseases in children.

Visitation of children with behavioural and attention disorders and learning disabilities.

Domain four

The health problems in relation to chronic disease, cancer and pathological aging.

Monitor, handle, maintain contact and adjust treatment in patients with chronic obstructive pulmonary disease, type 2 diabetes, ischemic heart disease, hypertension, asthma, myxoedema/thyrotoxicosis, rheumatologic disorders, osteoporosis, dementia, stroke and obesity. Be able to take part in interdisciplinary cooperation about the chronic patient,

Evaluate and handle patients with functional symptoms to prevent chronicity.

Recognise disease patterns of both single and multiple organ type that cannot be better explained by traditional somatic or psychiatric disease, and could diagnose health anxiety.

Take part in the treatment regime of patients with cancers diseases.

Primary communication with patients suffering from symptoms of possible malignant disease

Handle terminally sick patients. Including: Establish, be a part of and possibly lead an interdisciplinary cooperation concerning terminal patients who are in their own home.

Handle dying patients’ physical, mental, social and existential needs.

Handle palliation of the frequent symptoms in a terminal patient.

Know the special conditions that characterise elderly patients, with focus on: loss of function, polypharmacy, pain treatment, constipation, incontinence, visual and hearing impairment and grief and

sadness.
Deal with the confused and delirious patient with focus on diagnosis of depression, dementia, somatic deliria, confusion induced by medicine or alcohol.
Apply relevant psychometric test.

Domain five

The necessary non-medical expert skills and health promotion.

Communicate with patients and their relatives irrespective of social group and ethnicity**.**
Cooperate with governmental, municipal and regional authorities including institutions.
Illustrate knowledge of municipality and region structure, institutions and functions.
Demonstrate ability and willingness to act as coordinator/key person for patients, including leading and coordinating patient care. Recognise the role and responsibilities of the GP.
Know the importance of cultural, religious and ethnic matters in children's development and in the understanding of disease.
Apply clinical guidelines. The guideline must be able to be individualised, regarding the patient. The guideline must be able to be individualiennd functions..atic deliri

Demonstrate understanding of the fact that lifelong learning and development is a condition to clinical actions as a GP, including understanding the impact of own emotions, behaviour and ethics, as significant factors in order to safeguard a balance between work and private life.
Understand the need to prioritise time and resources for personal and professional development, and that such prioritisation is needed to ensure qualified medical professionalism, whilst counteracting burnout.
Plan own training.
